# Antibacterial and Antioxidant Activities of *Prinsepia utilis* Royle Leaf and Seed Extracts

**DOI:** 10.1155/2022/3898939

**Published:** 2022-10-17

**Authors:** Rakshya Bagale, Srijana Acharya, Akriti Gupta, Pooja Chaudhary, Gautam Prasad Chaudhary, Jitendra Pandey

**Affiliations:** Department of Pharmacy, Crimson College of Technology, Pokhara University, Devinagar-11, Butwal 32900, Nepal

## Abstract

Our study was designed to screen the antibacterial potency of *Princepia utilis* leaf and seed extract and to measure their antioxidant effects, total phenol content, total flavonoid content, and total carbohydrate content. Collected samples were extracted by cold maceration. Hexane, ethyl acetate, methanol, and distilled water were used as extraction solvents. In the disc diffusion method, *P. utilis* ethyl acetate leaf extract was most prominent against *Staphylococcus epidermis* with a zone of inhibition (ZOI) of 13.83 mm. Similarly, methanolic leaf extract was most prominent against *Staphylococcus aureus* (ZOI-12.33 mm). Furthermore, the methanolic seed extract was most sensitive against Klebsiella pneumoniaee (ZOI-11.66 mm) *Escherichia coli* (ZOI-9.0 mm). The lowest minimum inhibitory concentration (MIC) and minimum bactericidal concentration (MBC) values of 0.5 mg/mL and 0.6 mg/mL, respectively, were shown by ethyl acetate leaf extract against *S. epidermis*. Similarly, the highest values of MIC and MBC, i.e., 20.8 mg/mL and 33.3 mg/mL, respectively, were shown by hexane leaf extract against *S. epidermidis.* On the other hand, evaluation of antioxidant capacity revealed that ethyl acetate leaf extract showed the maximum antioxidant effect (IC_50_: 66.69 *μ*g/mL). The total flavonoid contents of different extracts were measured in the range of 37 ± 0.74 *μ*g QE/mg dry extract weight (methanolic seed extract) to 321.84 ± 4.82 *μ*g QE/mg dry extract weight (hexane seed extract). Likewise, *the total polyphenol content ranged from the* hexane leaf extract (17.33 ± 0.642 *μ*g GAE/mg dry extract weight) to ethyl acetate leaf extract (62.56 ± 1.284 *μ*g GAE/mg dry extract weight). We found a variation in total carbohydrate content in the range of 23.55 ± 1.125 *μ*g glucose/mg dry extract weight (hexane leaf extract) to 96.63 ± 2.253 *μ*g glucose/mg dry extract weight (aqueous leaf extract). Overall, this study revealed that leaf and seed extract of *P. utilis* exhibited noteworthy antibacterial effects against diverse pathogenic microorganisms.

## 1. Introduction

The study of plants is a significant way of developing raw materials for traditional as well as modern medicine [1]. Plant-derived medicine is essential for human physiology with its pharmacological action and has comparatively fewer side effects [2]. Primary and secondary metabolites are the major sources of phytomedicine [3]. Primary metabolites such as lactic acid, amino acids, nucleosides, enzymes, and coenzymes are crucial to life processes. Secondary metabolites like alkaloids, tannin, terpenoids, and flavonoids are derived biosynthetically from the primary metabolites [2, 4]. Bioassay-guided screening of medicinal plants in which particular therapeutic activity is targeted has resulted in the exploration and revelation of clinically beneficial drug molecules to combat life-threatening human diseases [1, 5–7]. Since ancient times, Ayurveda has always given major emphasis on the utilization of plant-derived extracts and herbal nutritional supplements to treat diverse infectious diseases as complementary or alternative medicines [8]. The discovery and development of modern allopathic medicine have been proven to be the most effective for mitigating a diverse range of pathological conditions. However, because of their high price, unwanted side effects, and difficulty for accessibility, many patients feel convenient to consume plant-derived products for remedy [8, 9]. In the present situation, infectious diseases caused by bacterial strains are becoming a threat throughout the world because of an increase in their resistance to modern antibiotics. This situation has ultimately resulted in the evolution of multi-resistant microorganisms [9]. Thus, the trajectory of morbidity and mortality is on an upward trend due to the unavailability of long-lasting effective medicines and the skyrocketing cost of new generation antibiotics [10]. Despite the increased rate of microbial resistance, the discovery and optimization of disease-specific antimicrobial medicine are in doubt. This calamitous circumstance has motivated us to search for more efficacious antimicrobial drugs by utilizing plant material so that these herbs will serve as major sources of active therapeutic molecules as as will help to obtain lead molecules for the synthesis of optimized new drug molecules [11].

**Figure 1:**
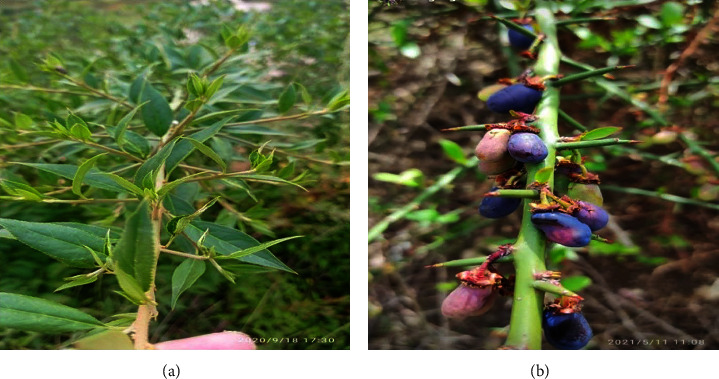
*P. utilis* plant: (a) Fresh leaf; (b) Ripen fruit.

*P. utilis*, which belongs to the family Rosaceae, is a medium-sized deciduous shrub (Figure 1), native to the Himalayan area and distributed up to an altitude of 1600–3000 m, commonly in India, China, Nepal, Taiwan, and Bangladesh [12, 13]. In Nepal, this plant is widely distributed in the western and central districts like Dadeldhura, Mugu, Humla, Jumla, Jajarkot, Kalikot, Myagdi, Lamjung, and Makawanpur, at high altitudes of 1800–300 m [14–17]. In Nepal, this plant is famous for the name “Gotyalo” or “Dhatelo” [12]. ETHNOMEDICALLY, the most significant asset of this plant is the seed, which constitutes approximately 37.2% fat [18]. In the remote areas of Nepal and India, the *P. utilis* seed oil is utilized as food cooking oil as well as a massage oil for headaches, boils, rheumatism, acne, stomach pain, muscular pain, skin infection, during pregnancy for easy delivery, and sedative purposes [19]. The paste of the oil cake is very useful for curing eczema or ringworm. Warm seed oil is effective to treat the body ache, caused due to heavy physical load. This warm oil also acts as a natural emollient for cracked feet and hands in the winter season [14]. The root bark is used for stomach disorders [13] and arthritis [12]. Besides that seed oil and leaf of *P. utilis* are traditionally used in treating arthritis, bone disorders, joint ailments, blood pressure, and atherosclerosis [12, 20].

Aside from the traditional uses, various scientific experiments have illustrated that this plant possesses diverse pharmacological effects such as antiinflammatory effect by its fruit [21], immunosuppression action of leaf [22], hypoglycemic [23] and antibacterial effect [24] of fruit, cytotoxic effect from aerial parts [25], digestive enzymes inhibitory effect from fruit [26], *α*-glucosidase inhibitory effect [27], pancreatic lipase inhibitory effect [27], and tyrosinase inhibitory effect [27] by its flower, etc. Furthermore, diverse pharmacologically active compounds such as hydroxylnitrile-glucosides, rutin, diterpenoidglucosides, isorhamnetin-3-*O*-rutinoside, cyanidin-3-O-rutinoside, triterpenoids, quercetin-3-*O*-glucoside, pentacyclictriterpenoids, etc have been isolated from the *P. utilis* [25, 26]. Also, *P. utilis* seed oil has a long history of ethnomedicine and economic importance to different ethnic groups in Nepal [13, 14]. After in-depth study of scientific literature about *P. utilis*, we came to know that investigation into the antibacterial activity of its leaves and seed oil has not been conducted yet. Therefore, this exploration was focused on the determination of the antibacterial effect of leaf and seed extract of *P. utilis* and examining their prospective antioxidant activity, total polyphenol, total flavonoid, and carbohydrate content along with preliminary phytochemical screening.

## 2. Materials and Methods

### 2.1. Chemicals and Reagents

Ciprofloxacin and gentamicin (Microxpress, a division of Tulip Diagnostics Pvt. Ltd.) antibiotic discs were utilized as standard drugs for antibacterial assay. Nutrient broth (HiMedia Laboratories Pvt. Ltd., Mumbai), Mueller Hinton Agar (MHA) (HiMedia Laboratories Pvt. Ltd., Mumbai), 3 (4, 5 dimethylthiazol-2-yl)-2-5-diphenyl tetrazolium bromide (MTT) (Beyotime Biotechnology, China), DPPH (Thermo Fisher scientific, India Pvt. Ltd; Mumbai), Barium chloride (HiMedia Laboratories Pvt. Ltd., Mumbai), Folin-Ciocalteu reagent, quercetin, ascorbic acid dimethyl sulfoxide (Thermo Fisher Scientific, India Pvt. Ltd., Mumbai).

### 2.2. Bacterial Strains

To explore the *in vitro* antibacterial activity of all the collected plant materials, gram-negative bacteria; *E. coli* (ATCC 14948), *K. pneumoniae* (ATCC 4352), and gram-positive bacteria; *S. epidermidis* (ATCC 12228), *S. aureus* (ATCC 9144) were obtained from Medicross Laboratory, Butwal, Nepal.

### 2.3. Plant Samples

Healthy and mature fruits of the *P. utilis* Royle and leaves (Figure 1) were collected with the help of local people of Narakot, Jumla (a temperate region, 3128 m above sea level) in November 2020. The collected plant materials were identified and authenticated by the National Herbarium and Plant Laboratory in Godabhari, Nepal (Ref no.078/079). The preparation and preservation of the herbarium were carried out in the Pharmacognosy Laboratory of the Crimson College of Technology, Nepal (Herberium number: CCT/HRB/2021-012). Collected and authenticated leaves and seeds were washed, dried, and ground to a fine powder. The fine powder mass was shifted through the (40 mesh size).

### 2.4. Preparation of the Plant Extracts

**Figure 2:**
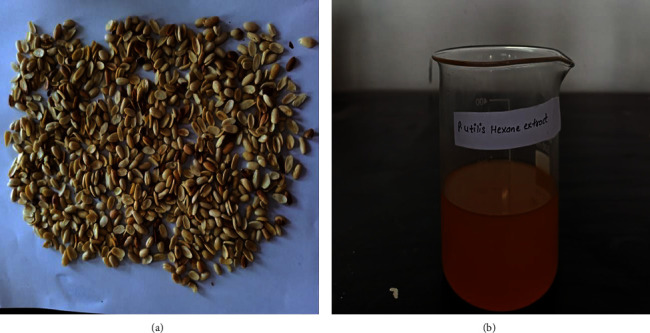
*P. utilis* plant samples. (a): dried seed kernels; (b) hexanolic extract of seed kernel.

Initially, the collected leaf and ripened fruits were cleaned by using distilled water. The ripened fruits of *P. utilis* were separated into a pulp and hard seed. Seed kernels (Figure 2) were removed from the hard shell. The leaf and healthy seed kernels were dried over the clean filter paper in the well-ventilated laboratory room at 25°C for two weeks. After the completion of the drying process, the dried leaves were well comminuted by using a grinder to a fine powder followed by sieving through the mesh sieve (40 mesh size). The dried seeds were also ground to achieve a sticky coarse powder. Both leaves and seeds were then subjected to cold maceration.

A triple cold maceration process was employed for all plant parts to achieve the optimal extraction. After single maceration with occasional manual shaking (every 6 hours for 3 days), the menstruum was collected and marc was further extracted with the same amount of fresh solvents. The whole procedure was repeated up to three times. Specifically, 100 g each of seed and leaf of *P. utilis* were extracted with 1000 mL of water, methanol, ethyl acetate, and hexane. The liquids from each step of maceration were strained, filtered, mixed, and dried at 40°C to obtain a gummy concentrate with the help of a rotatory evaporator. All the extracts were preserved in the refrigerator at 4 ± 1°C.

### 2.5. Extractive Yield Value

The extractive yield of *P. utilis* seed *and* leaf in different solvent was calculated with the help of the following equation:

(1) $$\begin{array}{c} Extractive\;yield=\frac{Amount\;of\;the\;extract\;obtaine\;d\left(g\right)}{Amount\;of\;crude\;sample\;used\;during\;extraction\left(g\right)}\times 100\text{.} \end{array}$$

### 2.6. Phytochemical Screening

Phytochemical screening of all the extracts was carried out to investigate the presence of diverse bioactive secondary metabolites, namely, polyphenols, alkaloids, phytosterols, cardiac glycosides, saponin, anthraquinone, resin, tannin, flavonoid, and terpenoids, with the help of different standard methods described in literature [28–30]. The presence was denoted by +sign and absence was denoted by −sign.

### 2.7. Determination of Total Phenolic Content

Total phenolic content was determined by using the Folin-Ciocalteu method with slight modification [31]. Gallic acid was used as a standard. In this study, different concentrations of gallic acid (3.906 *μ*g/mL, 7.812 *μ*g/mL, 15.625 *μ*g/mL, 31.250 *μ*g/mL, 62.500 *μ*g/mL, 125 *μ*g/mL, and 250 *μ*g/mL) were prepared by serial dilution technique. For each sample, extract solution of 1 mg/mL concentration was prepared by using ethanol. Then, 1 mL (1 mg/mL concentration) of ethanolic extract solution was treated with 1 mL (2°N) FC reagent followed by the addition of 5 mL distilled water and shaken well for 5 minutes. After that, 1 mL of 10%, Na_2_CO_3_ was poured and incubated for 1 hour. In the same manner, a blank solution was prepared without a sample. Finally, the absorbance of incubated standard solutions and sample solutions was recorded at 725 nm. All the measurements were evaluated in triplicate [24].

### 2.8. Determination of Total Flavonoid Content

The total flavonoid content was determined by using the method described in the literature [31]. Quercetin was used as a standard. Different concentrations of quercetin (31.25 *μ*g/mL, 62.50 *μ*g/mL, 125 *μ*g/mL, 250 *μ*g/mL, 500 *μ*g/mL, and 1000 *μ*g/mL) were prepared by the serial dilution technique. For each sample, extract solution of 1 mg/mL concentration was prepared by using ethanol. Then, 1 mL (1 mg/mL concentration) of ethanolic extract solution was well mixed with 4 mL of distilled water and 0.3 mL of 5% NaNO_2_. After continuously shaking for 5 minutes, 0.3 mL of 10% AlCl_3_ was added and subjected to incubation for 5 minutes. Then, 2 mL of 1 M NaOH was added to the solution. In the same manner, a blank solution was prepared without a sample. The final mixture solution was then incubated at room temperature for approximately 30 minutes. Finally, the absorbance of incubated standard solutions and sample solutions was recorded at 415 nm. All the measurements were evaluated in triplicate.

### 2.9. Determination of Total Carbohydrate Content

The total carbohydrate content was determined by using the phenol sulphuric acid method [31]. In this test, glucose was taken as a standard. Different concentrations of glucose (16.125 *μ*g/mL, 32.250 *μ*g/mL, 62.500 *μ*g/mL, 125 *μ*g/mL, and 250 *μ*g/mL) were prepared in distilled water by serial dilution technique. For each sample, the extract solution of 1 mg/mL concentration was prepared by using distilled water. Then, 2 mL of the aliquot sample was treated with 1 mL of 5% phenol solution. After that, 5 mL of conc. H_2_SO_4_ was added to the mixture shaking properly. After well shaking of mixture solution up to 10 minutes, it was kept in a water bath (30°C) for 20 minutes. In the same manner, a blank solution was prepared without a sample. Finally, the absorbance of incubated standards solutions and sample solutions were recorded at 490 nm. All the measurements were evaluated in triplicate [24].

### 2.10. Antioxidant Activity Determination by DPPH Free Radical Scavenging Method

The antioxidant activity of plant extract was examined by using DPPH (1, 1-diphenyl-2-picrylhydrazyl) free radical scavenging activity [31, 32]. First, the stock solution of 0.1 mM of DPPH, 1 mg/mL of ascorbic acid, and test solutions were prepared in ethanol. The ascorbic acid solution thus prepared was diluted into different concentrations (1 *μ*g/mL, 2.5 *μ*g/mL, 5 *μ*g/mL, and 10 *μ*g/mL). For the DPPH free radical inhibition assay, 4 mL of different extract solutions (500 *μ*g/mL, 250 *μ*g/mL, 125 *μ*g/mL, 62.50 *μ*g/mL, 31.25 *μ*g/mL, and 15.625 *μ*g/mL) of the samples were mixed with the same volume of DPPH solution (0.1 mM) and incubated in a dark place for about 30 minutes. After that, the absorbance of the sample mixture was monitored at 517 nm with the help of a UV spectrophotometer. Ethanol and ascorbic acid were chosen as blank and positive controls, respectively. All the measurements were examined in triplicate. The free radical scavenging activity was expressed as an inhibition percentage and was calculated using the following formula:

(2) $$\begin{array}{c} Percentage\;radical\;scavenged=\left[\frac{\left(A_{0}-A_{S}\right)}{A_{0}}\right]*100\%\text{,} \end{array}$$

where *A*_0_ was the absorbance of the control and *A*_*S*_ was the absorbance of the sample. The minimum inhibitory concentration (IC_50_), which signifies the minimum concentration that can completely scavenge 50% of the DPPH free radical, was also calculated through the Microsoft excel trend method.

### 2.11. Evaluation of Antibacterial Activity

#### 2.11.1. Preparation of Filter Paper Discs and Plant Extracts

For every extract, 100 mg was taken accurately in a small vial and dissolved homogeneously in 1 mL of DMSO, followed by storage of samples until use. The concentration of the extract solution was prepared in such a way that each 10 *μ*L of sample solution carried 1 mg of extract. About 5 mm diameter of filter paper disc was prepared by using Whatman's No. 1 filter paper and sterilized at 115°C for up to 15 minutes. [33–36].

#### 2.11.2. Preparation of Muller Hinton Agar (MHA) Media and Bacterial Strains Sub-Culturing

A widely accepted disc diffusion method was adopted to investigate the antibacterial activity of all the plant extracts obtained. For this, about 38 g of MHA was poured into a 1000 mL conical flask, and 1000 mL of distilled water was added to it. After completely dissolving the media, the conical flask was closed with a cotton plug and aluminum foil. After that, the flask was subjected to sterilization in an autoclave for 15 minutes at 121°C and 15 lbs pressure. The sterile conical flask media was placed for cooling to cool to 40–50°C in laminar airflow (sterilized). The media was carefully poured into each Petri plate and allowed to set. Two hardened media were kept for incubation at 37°C for 1 day to check the possible contamination, and the remaining samples were refrigerated at 5°C. To perform sub-culturing, the inoculating loop was inflamed, cooled, and dipped inside the tube to carry up the desired bacteria. Then, the loop was gently smeared all over the surface of the agar plate in a zigzag pattern. In this way, all the test organisms were subcultured by using individual sterilized agar plates with appropriate labeling. The subcultured plates were then subjected to incubation at 37°C for 24 hours before inoculation. The entire experiment was completed in aseptic conditions with laminar airflow.

#### 2.11.3. Bacterial Suspension/Inoculum Preparation

Firstly, nutrition broth media was prepared and kept for sterilization. Then, about 5 mL of nutrient broth was poured into four different sterilized test tubes, which were labeled with the name of the individual bacterial strain. From previously sub-cultured media, each bacterium was transferred into well-label respective tubes with the help of an inoculating loop. Finally, all the broth media containing bacteria were kept for incubation at 37°C for 1 day to achieve the suspensions of *K. pneumoniae*, *E. coli*, *S. aureus*, and S. epidermidis. The turbidity of the inoculum suspension was compared and adjusted up to the turbidity of 0.5 McFarland solutions.

#### 2.11.4. Screening and Measurement of Zone of Inhibition (ZOI)

For the ZOI calculation, a cotton swab stick (sterile) was submerged into the turbidity-adjusted bacterial suspension test tube. Then, inoculation of bacteria on an agar plate was achieved by rubbing the bacteria-loaded cotton swab over the entire surface of the sterile medium. A similar process was repeated for every bacterial strain. After that, the total area of media plates was roughly divided into four equal parts to keep the commercial standard antibiotic disc and filter paper disc (containing blank control and sample extract), at an equivalent distance. A 10 *μ*g/disc of gentamycin and ciprofloxacin was used as a positive control for gram-positive and gram-negative bacteria respectively. Exactly 10 *μ*L of each extract solution was transferred into two paper discs (doublet manner) to load the 1 mg of extract per disc. The third paper disc was loaded with 10 *μ*L DMSO as a blank control. All the plates were placed for incubation at 37°C for 24 hours. All the experiments were done in a triplicate manner. After incubating for 24 hours, all the culture media were examined for the determination of the inhibited area of bacterial growth (ZOI) by the standard antibiotic and different extracts, by utilizing a digital Vernier Caliper.

#### 2.11.5. Determination of MIC and MBC

The MIC and MBC of all the plant extracts against four different bacterial strains were determined with the help of a two-fold serial broth microdilution technique. For each extracted sample, a total of 10 well-labeled vials were sterilized, and then 750 *μ*L of sterilized Mueller-Hilton Broth (MHB) was poured into each. For the preparation of 10 different sample solutions of diluted concentrations (200 mg/mL–0.390625 mg/mL), 200 mg/mL of stock solution was prepared in DMSO and subjected to serial dilution by utilizing a 1 : 1 mixture of DMSO and water. After that, 250 *μ*L of sample solution was micro-pipetted into a respective vial contining 750 *μ*L of MHB, so that the ultimate concentration of sample solution ranged from 50 mg/mL to 0.09765 mg/mL. Bacteria with an inoculum of about 1 × 10^5^ CFU/mL were loaded into each vial. To prepare an inocula of microorganisms, broth culture was kept for incubation for 12 hours, and the turbidity of the suspension was adjusted to a similar to that of 0.5 McFarland standards. Also, one inoculated vial was incubated for the negative control, to the confirm desired sterility of the broth for microorganism growth. Also, 4% DMSO was used as a blank control. After that, all the samples were incubated for 24 hours at 37°C. Finally, the MIC values were calculated. MIC was considered as the minimum concentration of the extract that prohibited visible bacterial culture growth. The viability of the bacterial cells was checked with the help of 3(4, 5 dimethylthiazol-2-yl)-2-5-diphenyl tetrazolium bromide (MTT) by incubating at 37°C for a further 2 hours and visual inspection of formazan formation.

The MBC was determined to identify the lowest plant extract concentration that could completely kill the investigated bacteria. For this, the refrigerated MHA Petri plates were incubated at 37°C for 45 minutes and transferred into the sterilized laminar airflow (LAF) hood. After that, samples from each diluted test tube, which were prepared in the same manner as in MIC evaluation, were subcultured again on MHA plates. Then, sub-cultured media were kept for incubation at 37°C for 24 hours. Finally, the minimum concentration of plant extract that completely prevented bacterial growth over the media surface was considered the MBC.

### 2.12. Statistical Analysis

All the experiments were analyzed three times, and the results were presented as mean ± SD. The statistical significance of differences was calculated by one-way ANOVA and Tukey's test.

## 3. Results and Discussion

### 3.1. Extractive Yield Value

The extractive yield values of *P. utilis* leaf and seed in water, methanol, ethyl acetate, and hexane were calculated by using equation (1) and the results are depicted in Table 1.

### 3.2. Phytochemical Screening

A qualitative examination of phytochemicals is a crucial step to collecting scientific information about the occurrence of pharmacologically significant secondary metabolites in plants, disclosing a vital role towards in the useful physiological and medicinal properties such as antiviral, anticancer, antimicrobial, antidiabetic, antioxidant, and antihypertensive activities [37–39]. Phytochemical screening of leaf and seed extract of *P. utilis* in various solvents displayed the presence of different amounts of alkaloid, saponin, terpenoid, anthraquinones, tannin, cardiac glycoside, flavonoid, carbohydrate, polyphenol, protein and amino acid, resin, and phytosterol presence. In our study, all the seed extracts were alkaloid and anthraquinone-free. Cardiac glycosides were only detected in polar fractions, i.e., aqueous leaf extract and methanolic seed extract. Moreover, higher contentrations of phytochemicals were reported in methanolic and aqueous extracts of both samples. From several scientific studies, it has been proven that *P. utilis* leaf extract contains various bioactive compounds such as p-Coumaric acid, rutin, catechin, Kaempferol-3-O-glucoside, Quercetin-3-O-glucoside, Isorhamnetin-3-O-glucoside, Isoschaftoside, etc. [27]. Besides that, various bioactive terpenoids such as epiutililactone, utililactone, 2*α*-O-trans [1]p-coumaroyl-3*β*, 19*α*-dihydroxy-urs-12-en-28-oic acid, 2*α*-O-cis-p-coumaroyl-3*β*, 19*α*-dihydroxy-urs-12-en-28-oic acid, and coumarin derivatives have been reported from this plant [22, 25]. Moreover, *P. utilis* seed extract was reported to contain hydroxylnitrile-glucosides compounds (prinsepicyanosides *A*-*E*) [40]. The results for the preliminary phytochemical screening are summarized in Table 2.

### 3.3. Determination of Total Phenolic Content

Polyphenol compounds are profusely available phytoconstituents in plant species. One or more hydroxyl groups found in polyphenol compounds tend to scavenge any free radicals encountered. Hence, there is a direct correlation between the total polyphenol content and the antioxidant capacity of many plant species. There is some scientific share of evidence that phenolic compounds can donate hydrogen efficiently and can serve as very good antioxidant molecules [41]. In our research, the quantitative evaluation of total phenolic content was achieved with the utilization of Folin-Ciocalteu reagent, and all the values were expressed as gallic acid equivalent (GAE)/mg of dry extract.  Table 3 provides information about the total phenolic content of different *P. utilis* extracts in terms of *μ*g gallic acid equivalent/mg dry extract. Among the seven different extracts, we found a significant variation of total phenol content ranging from hexane leaf extract (17.33 ± 0.642 *μ*g GAE/mg dry extract weight) to ethyl acetate leaf extract (62.56 ± 1.284 *μ*g GAE/mg dry extract weight). By analyzing the data from Table 3, it is clear that the extracting solvent also has a significant influence on the extraction of the phenolic content of the different plant parts. Furthermore, we can observe significant variation among different parts of the plant, within the same solvent. The statistical analysis also displayed a significant difference (*p* < 0.05) in the total phenolic content when each part was compared with different solvents as well as when different parts were compared with the same solvent. It is quite obvious that the significantly maximum total phenol content was observed in the ethyl acetate leaf extract and the significantly lowest amount of total phenol content was noted in the hexane leaf extract. In a previous study, the total phenolic content of *P. utilis* seed was reported to be 81.43 mg GAE/100 g of dried seed powder [42]. Also, another study reported that the hydroalcoholic extract of *P. utilis* leaf contained 23.48 GAE/mg dry extract [27]. Moreover, diverse classes of flavonoid compounds from the fruit [26] and leaf extract [27] have been reported in previous research papers.

### 3.4. Determination of Total Flavonoid Content

Flavonoids are a highly multiform and extensive class of natural phenolic compounds. Hydroxyl positions present in the flavonoid compounds can display antioxidant activity and it depends on the hydrogen or electron donation tendency of the flavonoid to a free radical [43]. In our investigation, seven different extracts of *P. utilis* were quantitatively examined for the calculation of total flavonoid by precipitating the extract solution with aluminum chloride (AlCl_3_), with the help of an alkalinized medium. Data for the total flavonoid content in different extracts are presented in Table 3. Among seven different samples, we found a variation in total flavonoid content in the range of 37 ± 0.74 *μ*g QE/mg dry extract weight (methanolic seed extract) to 321.84 ± 4.82 *μ*g QE/mg dry extract weight (hexane seed extract). By observing Table 3, we can be sure that the solvent used for the extraction has a prominent role in the isolation of flavonoid content from the different parts of the plants as well as each plant part (leaf and seed) has different content even in the same solvent. The statistical analysis showed a significant difference (*p* < 0.05) in the total flavonoid content when each part was compared in different solvents as well as when different parts were compared in the same/each solvent. In our investigation, the order of the flavonoid content in different samples of P. utilis is as follows; seed > leave. Among the different solvent extracts of the leaf samples, the maximum flavonoid content was reported to be in ethyl acetate leaf extract, i.e., 269.12 ± 4.08 *μ*g QE/mg dry extract. In the same way, the maximum amount of flavonoid among the different solvent extracts of seed was represented by the hexane seed extract, i.e., 321.84 ± 4.82 *μ*g QE/mg. In a previous study, the total flavonoid content of *P. utilis* seed was reported to be 226.92 mg QE/100 g of dried seed powder [42]. Also, another study reported that the hydroalcoholic extract of *P. utilis* leaves contained 91.36 QE/mg dry extract [27]. Moreover, diverse classes of flavonoid compounds from the fruit [26] and leaf extract [27] have been reported in previous research papers.

### 3.5. Determination of Total Carbohydrate Content

Carbohydrates are a large group of primary metabolites which are copiously synthesized during the process of photosynthesis, and these classes of molecules serve as vital components of a plant cell. Carbohydrates are the vital source of energy that are required to govern the metabolic reactions, stimulate the secretion of insulin, serve as a crucial component of neurotransmitters, and adjust the concentration of serotonin in living organisms [44]. In our investigation, seven different extracts of *P. utilis* were quantitatively examined for the calculation of total carbohydrate content by using the Phenol Sulphuric method. As shown in Table 3 the carbohydrate contents from all the examined extracts were presented as *μ*g glucose equivalent per milligram dry extract weight. Among seven different samples, we found a variation in total carbohydrate content in the range of 23.55 ± 1.125 *μ*g glucose/mg dry extract weight (hexane leaf extract) to 96.63 ± 2.253 *μ*g glucose/mg dry extract weight (aqueous leaf extract). By analyzing the data from Table 3, it is clear that extracting solvent also has a significant influence on the extraction of the carbohydrate content of the different plant parts. Furthermore, we can observe significant variation among different parts of the plant within the same solvent. The statistical analysis also displayed a significant difference (*p* < 0.05) in the total carbohydrate content when each part was compared with different solvents as well as when different parts were compared with the same solvent. As shown in Table 3, a moderate amount of carbohydrate content was detected in all the investigated extracts. For the first time, the scientific information about the quantitative carbohydrate contents of all the samples was documented.

### 3.6. Antioxidant Activity Measurement by DPPH Free Radical Scavenging Method

**Figure 3:**
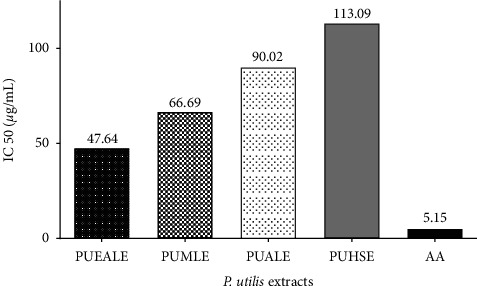
IC_50_ values of different extracts of leaf and seed of *P. utilis* extracts along with ascorbic acid (AA).

The hydrogen atom or electron donation potency of all the plant extracts to the DPPH free radical was examined by the bleaching of DPPH ethanolic solution. The DPPH radical can absorb UV radiation at wavelength of 517 nm. The radical scavenging capacity of different extracts of *P. utilis* was calculated by quantitatively monitoring the fall in absorbance [34]. Among seven different extracts from leaves and seeds of *P. utilis*, our investigation reported that ethyl leaf extract manifested the highest DPPH free radical inhibition of 98.14% at a concentration of 500 *μ*g/mL and the minimum free radical inhibition effect was displayed by methanolic seed extract (13.94 at 500 *μ*g/mL). Data for the free radical scavenging potency of the different extracts are depicted in Table 4. Among the seven different extracts, the best IC_50_ was shown by ethyl acetate leaf extract (47.64 *μ*g/mL) and methanolic leaf extract (66.69 *μ*g/mL). However, the hexane leaf extract, ethyl acetate seed extract, and methanolic seed extract could not inhibit 50% of the DPPH radical, within the examined concentrations. Data for the IC_50_ values of different extracts and ascorbic acid is represented in Figure 3. Overall, the DPPH free radical inhibitory action of *P. utilis* leaf and seed extracts was found to be moderate. Moreover, the good antioxidant value of hexane seed extract justified the ethnomedicinal use of *P. utilis* seed oil for edible and other medical purposes. Among different solvents, in the case of leaf extract, the most prominent inhibitory action was expressed by the methanolic extract. However, surprisingly, hexane extract revealed the most noticeable free radical scavenging effect in the case of *P. utilis* seed. On top of that, extracts with more flavonoid and phenolic contents were found to be capable of scavenging free radicals with high affinity. In a previous study, the hydroalcoholic extract of *P. utilis* leaf scavenged approximately 67% DPPH free radical at a concentration of 100 *μ*g/mL [27]. In another study IC_50_ of *P. utiis*, methanolic leaf extract was reported to be 62.46 *μ*g/mL [12], which is almost comparable to our data. Furthermore, in research conducted in China, about 63% of DPPH free radical was reported to be scavenged by dry seed powder of *P. utilis* at the concentration 20 mg dry seed/mL [42]. The DPPH free radical scavenging effect shown by standard sample ascorbic acid is described in Table 5.

### 3.7. Antibacterial Activity Analysis

A total of 7 different extracts obtained from the leaves and seeds of *P. utilis* were examined for its antibacterial activity against four different microbial strains. Their antibacterial activity was quantitatively determined by observing an inhibition zone presence or absence all over the disc, added with the extract. As shown in Table 6, most of the extracts are more effective against gram-positive bacterial strains as compared to gram-negative ones. Generally, plant extracts can show a more suppressive effect on gram-positive bacterial strains than gram-negative bacterial strains because of the presence of drug-resistant lipopolysaccharide composition in the multi-layered cell wall of gram-negative strains [45, 46].

In our study, *P. utilis* ethyl acetate leaf extract was found to be most prominent against *S. epidermis* (ZOI-13.83 mm). However, this extract was insensitive against *S. aureus*. Similarly, methanolic leaf extract was most prominent against *S. aureus* (ZOI-12.33 mm). Furthermore, the methanolic seed extract was most sensitive against *K. pneumoniae* (ZOI-11.66 mm) and *E. coli* (ZOI-9.0 mm). Also, the methanolic leaf extract was reported to be most sensitive against both gram-positive strains. Interestingly, the methanolic seed extract was successful in suppressing the growth of gram-negative as well as gram-positive bacterial strains. Our finding about the broad-spectrum activity of *P. utilis* seed extract was also supported by previous research conducted in China [24]. Between the two gram-positive bacteria, *S. epidermidis* was more sensitive than *S. aureus*, whereas *K. pneumoniae* was more sensitive than *E. coli* in the case of gram-negative bacteria. In summary, leaf extracts were more effective against gram-positive bacteria and seed extracts were more sensitive towards gram-negative bacteria. Previously published research studies about phytochemical analysis of *P. utilis* seed and leaf have shown that both parts are rich in diverse classes of polyphenols, flavonoids, and their glycosidic forms [26, 27]. However, a unique type of hydroxylnitrile-glucoside compound (prinsepicyanosides *A*-*E*) has been identified in *P. utilis* seed extract. These unique classes of bioactive compounds have shown excellent inhibitory effects against several gram-negative bacteria such as *Vibrio* parahaemolyticus, *Salmonella* gallinarum, *Vibrio* cholera, and *Vibrio* parahaemolyticus [40]. This might be the possible reason for the better inhibitory effect of *P. utilis* seed extract on gram-negative bacterial strains in our study.  Figure S1 shows the zone of inhibition formed by ethyl acetate leaf extract (Figure S1A) and methanolic leaf extract (Figure S1B) against *S. epidermidis*.

Out of a total of 28 samples against four different bacterial strains, a total of 15 samples exhibited measurable ZOI, which were further examined for the calculation of MIC and MBC. The values of MIC and MBC were expressed as mg/mL. The MIC and MBC values of different screened extracts were in the range of 0.5 mg/mL to 20.8 mg/mL and 0.6 mg/mL to 33.3 mg/mL, respectively. The lowest MIC and MBC values of 0.5 mg/mL and 0.6 mg/mL, respectively, were shown by ethyl acetate leaf extract against *S. epidermis.* However, this extract was ineffective against the *S. aures* for examined concentrations. In another hand, the highest values of MIC and MBC, i.e., 20.8 mg/mL and 33.3 mg/mL, respectively, were shown by hexane leaf extract against *S. epidermidis*. As shown in Table 7, only the methanolic seed extract was capable of inhibiting and killing all the tested microorganisms at the examined concentrations. Also, in the case of gram-negative bacteria, the lowest MIC and MBC value of 4.1 mg/mL and 6.2 mg/mL, respectively, were shown by methanolic seed extract against *E. coli,* and the highest values of MIC and MBC, i.e., 16.6 mg/mL and 20.8 mg/mL, respectively, were revealed by aqueous leaf extract against *K. pneumonia*e. All the results are presented in Table 7. Similarly, Figure S2 depicts the MBC shown by three different extracts against four different bacterial strains.

According to a study conducted by a researcher from China, *P. utilis* ethyl acetate seed extract was further fractionated by using normal phase column chromatography, to achieve seven different fractions. The antibacterial effect of each fraction was tested against *S. aureus* and *E. coli* by using the disc diffusion method. Among seven different fractions, fraction 1 (0.2 mg per disc) revealed measurable ZOI against *S. aureus* (14.16 mm) and *E. coli* (11.5 mm). The same fraction showed the lowest MIC and MBC values of 0.625 mg/mL and 2.5 mg/mL, respectively, against *S. aureus* whereas in the case of *E. coli*, the lowest MIIC and MBC values of 2.5 mg/mL and 5.0 mg/mL, respectively, were observed [24]. The better MIC and MBC values shown in that research in comparison to our study must be due to the examination of fractionated samples in that study. In another study, an alkaloid isolated from *P. utilis* has shown MIC values of 0.08 mg/mL and 0.625 mg/mL, against *S. aureus* and *E. coli*, respectively, whereas its MBC values against *S. aureus* and *E. coli* were 0.16 mg/mL and 1.25 mg/mL, respectively [47]. Although extensive studies on bioactive antimicrobial phytochemicals of *P. utilis* remain unexplored yet, few investigations have documented the occurrence of various potent bioactive compounds such as p-Coumaric acid, rutin, catechin, Kaempferol-3-O-glucoside, Quercetin-3-O-glucoside, Isorhamnetin-3-O-glucoside, Isoschaftoside, etc. [27]. Besides that, various bioactive terpenoids such as epiutililactone, utililactone, 2*α*-O-trans [1]p-coumaroyl-3*β*, 19*α*-dihydroxy-urs-12-en-28-oic acid, 2*α*-O-cis-p-coumaroyl-3*β*, 19*α*-dihydroxy-urs-12-en-28-oic acid, and coumarin derivatives have been reported from this plant [22,25]. Thus, these diverse bioactive components might be responsible for the significant antibacterial effect of *P. utilis* leaf and seed extract.

## 4. Conclusion

Overall, our present findings suggested that ethyl acetate and methanolic leaf extract of *P. utilis* manifested significant antibacterial and antioxidant effects. Also, hexane seed extract of *P. utilis* displayed moderate antioxidant activity with an appraisable amount of total flavonoid and phenol content to justify the ethnomedicinal use of *P. utlilis* seed oil. The presence of polyphenol and flavonoid components might be responsible for these biological activities. Therefore, this study concluded that *P. utilis* leaf extract can be recommended for the inhibition of gram-positive bacteria and seed extract can be recommended for the inhibition of gram-negative bacteria. Their notable antibacterial potency against four different pathogenic bacteria reflects that these extracts may serve as a useful source to combat various life-threatening pathogenic bacteria to mitigate various diseases like urinary tract infection, dental problems, dysentery, diarrhea, etc. Nevertheless, further advanced research activities with great priority on an animal model, along with an understanding of the mechanism of action of antibacterial activity, are mandatory to validate the traditional utilization of this medicinally significant plant and go after the long research-based expedition of medicinal plant-derived antibacterial medicine discovery, for ensuring human safety and protection of their health.

## Figures and Tables

**Table 1:** Percentage yield value from different crude extracts of *P. utilis* leaf and seed.

Plant pars	Solvent used	Total crude drug (g)	Obtained extract (g)	(%) Yield value
Leaf	Hexane	200	9.44	4.72
	Ethyl acetate	200	10.91	5.45
	Methanol	800	105.55	13.19
	Aqueous	200	18.62	18.16

Seed	Hexane	2000	528.71	26.43
	Ethyl acetate	200	8.08	4.04
	Methanol	79	40.50	51.27

**Table 2:** Phytochemical screening of *P. utilis* extracts.

Test	PUHLE	PUEALE	PUMLE	PUALE	PUHSE	PUEASE	PUMSE
Alkaloid	+	+	+	+	−	−	−
Saponins	−	+	+	+	+	+	+
Terpenoids	+	+	+	+	+	+	+
Anthraquinone	−	−	+	+	−	−	−
Tannin	+	+	+	−	+	+	+
Cardiac glycoside	−	−	−	+	−	−	+
Flavonoid	−	+	+	+	+	+	−
Carbohydrate	−	−	+	+	+	+	+
Polyphenol	−	+	+	+	−	−	+
Protein and amino acid	−	+	+	+	−	+	−
Resin	−	+	+	+	+	+	+
Phytosterol	−	−	+	+	+	+	+

Used in Table 2 are as follows: (PUHLE: *P. utilis* hexane leaf extract, PUEALE: *P. utilis* ethyl acetate leaf extract, PUMLE: *P. utilis* methanol leaf extract, PUALE: *P. utilis* aqueous leaf extract, PUHSE: *P. utilis* hexane seed extract, PUEASE: *P. utilis* ethyl acetate seed extract, PUMSE: *P. utilis* methanol seed extract).

**Table 3:** Results for the total phenol, flavonoid, and carbohydrate content of *p. uilis* leaf, and seed, extracted in different solvents.

Extracts	Total phenol content	Total flavonoid content	Total carbohydrate content
PUHLE	17.33 ± 0.64^a^	217.6 ± 2.90^a^	23.55 ± 1.13^a^
PUEALE	62.56 ± 1.28^b^	269.12 ± 4.08^b^	60.61 ± 1.41^b^
PUMLE	44.09 ± 0.29^c^	77.60 ± 3.42^c^	75.82 ± 0.43^c^
PUALE	26.57 ± 0.69^d^	104.57 ± 12.26^d^	96.63 ± 2.25^d^
PUHSE	21.59 ± 0.14^e^	321.84 ± 4.82^e^	51.17 ± 1.38^e^
PUEASE	35.74 ± 1.39^f^	288.21 ± 4.08^f^	39.28 ± 0.84^f^
PUMSE	49.05 ± 1.70^g^	37.00 ± 0.74^g^	78.88 ± 0.76^c^

All the data were represented as mean value ± standard deviation (*n* = 3). Different superscripts (*a*, *b*, c, *d*, e, *f*, *g*) within the column represent the significant differences (*p* < 0.05) among the contents of each part (leaf and seed) compared in different solvents (hexane, ethyl acetate, methanol, and water).

**Table 4:** DPPH free radical scavenging capacity of *P. utilis* leaf and seed extract obtained from different solvents at various concentrations.

Concentration (*μ*g/mL)	DPPH Scavenging activity (%)					
	15.625	31.25	62.5	125	250	500
PUHLE	7.13 ± 0.69	11.95 ± 0.42	15.40 ± 0.26	19.90 ± 0.32	23.59 ± 0.53	27.39 ± 0.31
PUEALE	28.85 ± 0.64	43.18 ± 0.64	53.39 ± 0.44	78.23 ± 0.44	87.84 ± 0.27	98.14 ± 0.87
PUMLE	25.43 ± 0.54	41.23 ± 0.89	55.57 ± 0.54	70.76 ± 0.17	86.05 ± 0.89	97.87 ± 0.17
PUALE	16.19 ± 0.30	31.35 ± 0.75	52.41 ± 4.12	71.84 ± 1.10	86.31 ± 0.52	87.30 ± 0.19
PUHSE	19.98 ± 0.61	28.04 ± 0.30	39.68 ± 0.19	68.51 ± 0.23	77.37 ± 2.56	90.81 ± 0.34
PUEASE	24.58 ± 0.27	25.09 ± 0.34	26.97 ± 0.34	30.18 ± 0.48	33.99 ± 0.52	36.10 ± 0.39
PUMSE	4.81 ± 0.21	7.66 ± 0.29	7.47 ± 1.09	7.66 ± 0.61	13.68 ± 0.19	13.94 ± 0.47

**Table 5:** DPPH inhibition percentage is shown by the standard sample ascorbic acid.

Concentration (*μ*g/mL)	(%) scavenged ± SD
10	92.45 ± 0.18
5	52.30 ± 0.30
2.5	26.98 ± 0.46
1.0	8.87 ± 0.08

**Table 6:** Zone of inhibition of leaf and seed of *P. utilis* extract in different solvent.

Zone of inhibition in mm (mean ± SD)				
Different sample	*E. coli*	*K. pneumoniae*	*S. epidermidis*	*S. aureus*
PUHLE	—	7 ± 0.00	10 ± 1.00	—
PUEALE	8 ± 0.00	7 ± 0.00	13.83 ± 0.28	—
PUMLE	—	6 ± 0.00	12.16 ± 0.28	12.33 ± 0.57
PUALE	—	10.33 ± 0.57	—	—
PUHSE	—	—	—	—
PUEASE	—	—	9 ± 0.00	10.16 ± 0.76
PUMSE	9 ± 0.00	11.66 ± 0.57	9 ± 0.00	11 ± 1.00
CIPROFLOXACIN	40.6 ± 1.10	24.66 ± 2.51		
GENTAMICIN			18.33 ± 0.57	18.33 ± 1.15
Negative controlled	—	—	—	—

*Note.* — indicates that extract is ineffective to inhibit bacterial growth in the evaluated concentrations.

**Table 7:** Determination of MIC and MBC values of leaf and seed extract of *P. utilis*.

MIC and MBC values of samples (mg/mL)								
Extract	*K. pneumoniae*		*E. coli*		*S. epidermidis*		*S. aureus*	
	MIC	MBC	MIC	MBC	MIC	MBC	MIC	MBC
PUMSE	8.3 ± 3.60	12.5 ± 0.00	4.1 ± 1.80	6.2 ± 0.00	1.3 ± 0.40	1.5 ± 0.00	10.4 ± 3.50	12.5 ± 0.00
PUEASE	—	—	—	—	5.2 ± 1.80	10.4 ± 3.60	20.8 ± 7.20	25 ± 0.00
PUEALE	8.3 ± 3.60	10.4 ± 3.60	5.2 ± 1.80	10.4 ± 3.60	0.5 ± 0.20	0.6 ± 0.20	—	—
PUMLE	12.5 ± 0.00	20.8 ± 7.20	—	—	2.6 ± 0.90	4.1 ± 1.80	10.4 ± 3.60	12.5 ± 0.00
PUALE	16.6 ± 7.20	20.8 ± 7.20	—	—	—	—	—	—
PUHLE	—	—	—	—	20.8 ± 7.20	33.3 ± 14.40	—	—

*Note.* — indicates that extract is ineffective to inhibit and kill the bacteria in the evaluated concentrations.

## Data Availability

All the data used to support the results of this research are available from Jitendra Pandey upon request.

## Supplementary Materials

Supplementary Materials Photographs of zone of inhibition (ZOI) calculation and minimum bactericidal concentration determination (MBC) are presented in Supplementary Figures S1 and S2. Figure S1: ZOI produced by *P. utilis* ethyl acetate leaf extract (A) and methanolic leaf extract (B), against S. epidermidis. Figure S2: MBC determination of *P. utilis* extracts (A: methanolic seed extract against *E. coli*, B: ethyl acetate leaf extract against *S. epidermidis*; C: ethyl acetate leaf extract against *K. pneumoniae* D: methanolic leaf extract against *S. aureus*).
