# Supplementary material for: Antibacterial and Antioxidant Activities of Prinsepia utilis Royle Leaf and Seed Extracts
Source: J Trop Med. 2022 Oct 17;2022:3898939. doi: 10.1155/2022/3898939 (PMC9592217; doi:10.1155/2022/3898939)
Supplement: Supplementary Materials — Photographs of zone of inhibition (ZOI) calculation and minimum bactericidal concentration determination (MBC) are presented in Supplementary Figures S1 and S2. Figure S1: ZOI produced by P. utilis ethyl acetate leaf extract (A) and methanolic leaf extract (B), against S. epidermidis. Figure S2: MBC determination of P. utilis extracts (A: methanolic seed extract against E. coli, B: ethyl acetate leaf extract against S. epidermidis; C: ethyl acetate leaf extract against K. pneumoniae D: methanolic leaf extract against S. aureus). [file 3898939.f1.pdf]

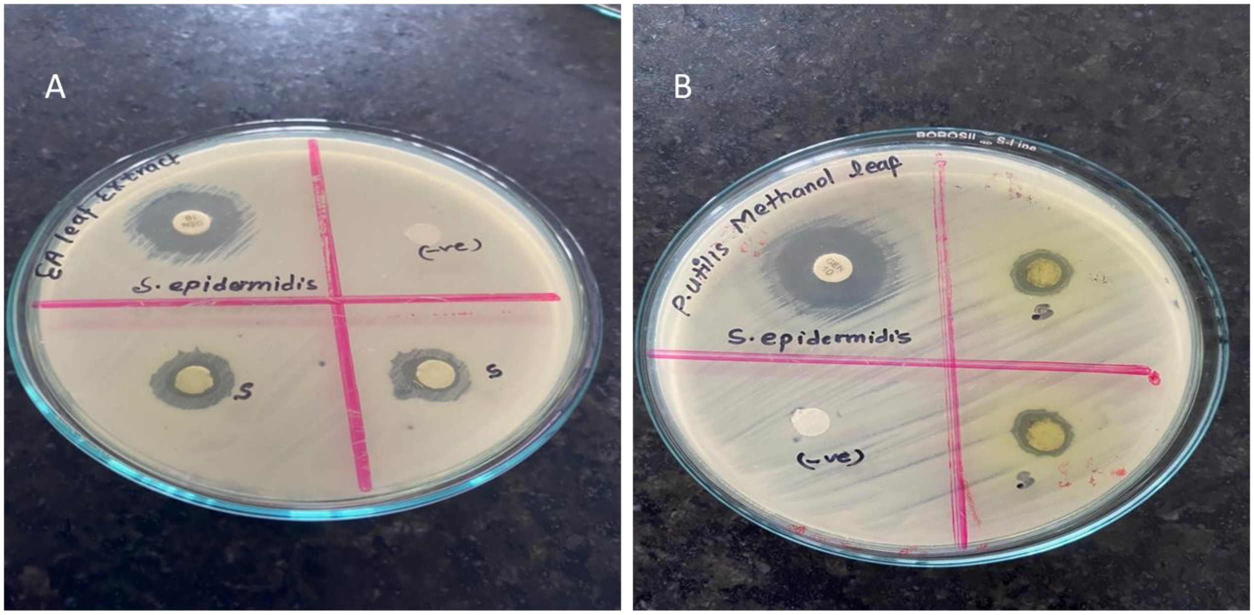

FIGURE S1: ZOI produced by *P. utilis* ethyl acetate leaf extract (A) and methanolic leaf extract (B), against *S. epidermidis*.

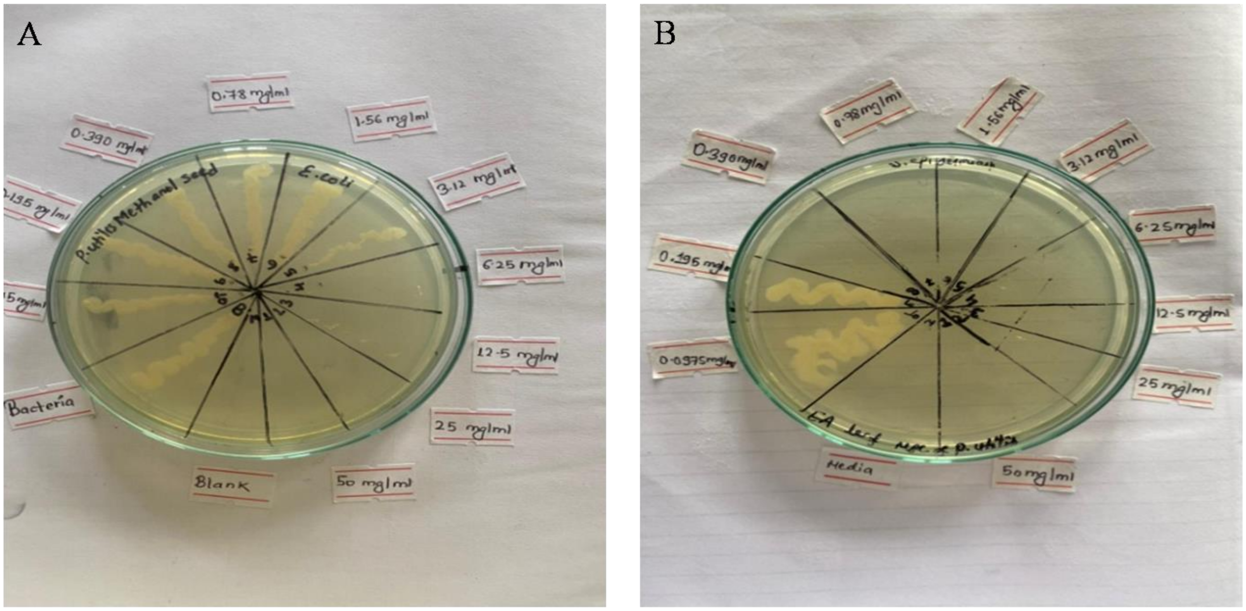

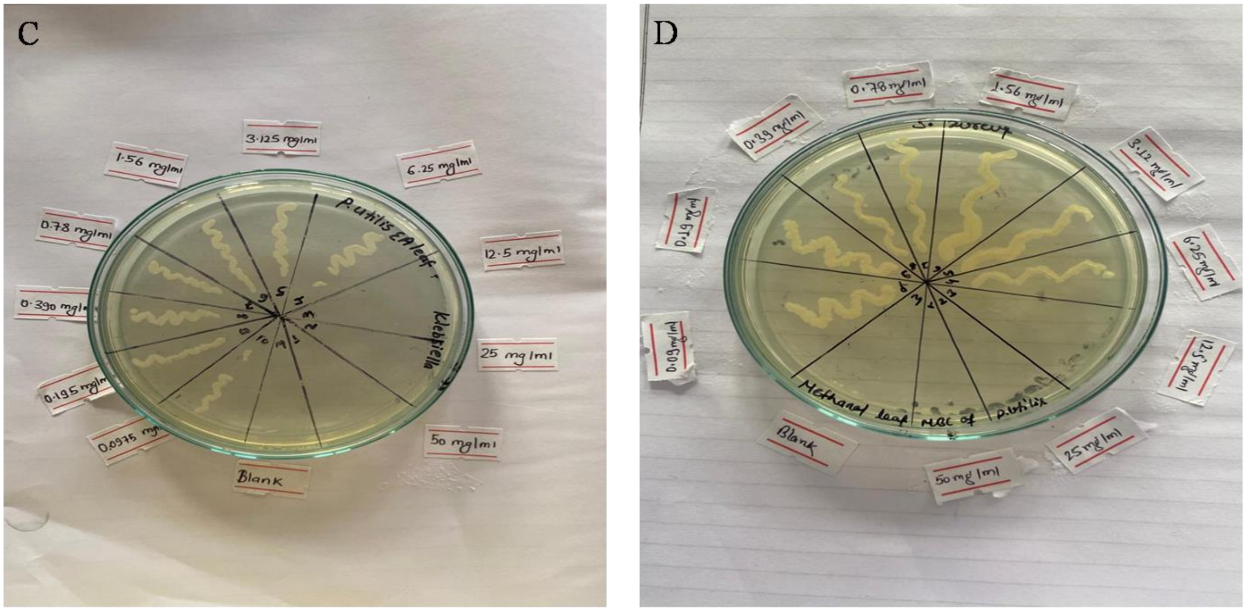

FIGURE S2: MBC determination of *P. utilis* extracts (A: methanolic seed extract against *E. coli*, B: ethyl acetate leaf extract against *S. epidermidis*; C: ethyl acetate leaf extract against *K. pneumoniae*; D: methanolic leaf extract against *S. aureus*).
